# Communicating bad news in oncology and hematology settings: A statistic and Large Language Model for interpreting nurses’ difficulties and emotions

**DOI:** 10.1017/S1478951525100771

**Published:** 2025-11-11

**Authors:** Elsa Vitale, Luana Conte, Marco Cioce, Patrizia Cornacchione, Angela Capuano, Raffaella Massafra, Ludovica Panzanaro, Giorgio De Nunzio, Matteo Steduto, Chiara Visintini, Sara Errichiello, Alfonso Parisi, Gabriele Sperti, Olga Pomes, Roberto Lupo, Stefano Botti

**Affiliations:** 1 Scientific Directorate, IRCCS Istituto Tumori “Giovanni Paolo II”, Bari, Italy; 2 Department of Physics and Chemistry, University of Palermo, Palermo, Italy; 3 Advanced Data Analysis in Medicine (ADAM), Laboratory of Interdisciplinary Research Applied to Medicine (DReAM), University of Salento and ASL (Local Health Authority) Lecce, Lecce, Italy; 4 Department UOC SITRA, Fondazione Policlinico Universitario A. Gemelli IRCCS, Rome, Italy; 5 UOC Fisica per le Scienze della Vita, Dipartimento di Diagnostica per Immagini e Radioterapia Oncologica, Fondazione Policlinico Universitario A. Gemelli IRCCS, Rome, Italy; 6 Department of Emergency, AORN Santobono-Pausilipon, Naples, Italy; 7 Psychiatric Department, C.R.A.P. Carrubo, Sol Levante srl, Avetrana, Taranto, Italy; 8 Laboratory of Biomedical Physics and Environment, Department of Mathematics and Physics “E. De Giorgi”, University of Salento, Lecce, Italy; 9 Haematological Intensive Care Unit, Fondazione IRCCS Casa Sollievo della Sofferenza, San Giovanni Rotondo, Italy; 10 Haematology and Stem Cell Transplantation Unit, Udine University Hospital, Azienda Sanitaria Universitaria Friuli Centrale, Udine, Italy; 11 Division of Hematology and Bone Marrow Transplant, Fondazione Istituto di Ricovero e Cura a Carattere Scientifico, Istituto Nazionale dei Tumori, Milan, Italy; 12 Department of Hematology and Bone Marrow Transplant, Hospital Card. G. Panico, Tricase, Italy; 13 University Cardiology Department, University Hospital Consortium Polyclinic of Bari, Bari, Italy; 14 San Giuseppe da Copertino Hospital, ASL (Local Health Authority) Lecce, Lecce, Italy; 15 Hematology Unit, Azienda USL-IRCCS of Reggio Emilia, Reggio Emilia, Italy

**Keywords:** Bad news, communication, generative artificial intelligence (GAI), oncology, hematology, setting

## Abstract

**Background:**

An effective communication seemed to be crucial in all the cancer care phases, like diagnosis, prognosis, and treatment options.

**Objectives:**

To analyze and interpret structured and open-ended questionnaire responses, focusing on the communication of bad news in onco-hematology: health care professionals’ attitudes, communication methods, and perceived stress levels.

**Methods:**

By employing a free Large Language Model, we identified and summarized the main emotions and perspectives shared by professionals.

**Results:**

A total of 221 Italian nurses and physicians employed in onco-hematology field were enrolled. The analysis revealed key emotional themes, offering insights into the professionals’ emotional states and coping mechanisms when delivering difficult news.

**Significance of results:**

Data highlighted the duality of emotions experienced by nurses when delivering bad news – balancing professional composure with emotional distress, underscoring the critical role of empathy, team support, and adequate preparation in helping nurses navigate these challenging conversations.

## Introduction

Health care professionals, particularly nurses and physicians, represent an essential role in guiding cancer patients and their related families through their cancer pathway. As highlighted from literature, nurses, and physicians dealt with all the continuum journey of care, beginning from health promotion to cancer prevention, to handle care, to cure until to palliative care (Vitale et al. 2021a; Nahm et al. 2023). Due to the continuity of contact that nurses and physicians have with their patients and families, they were in an optimal position to adopt essential role in health care delivery pathways (Hajizadeh et al. 2021). Cancer patients and their families reported high levels in psychological stress requiring emotional and social help (Lupo et al. 2021; Vitale 2022). Thus, an effective communication seemed to be crucial in all the cancer care phases, like diagnosis, prognosis, and treatment options (Wang et al. 2024).

Positive effects for an effective communication among cancer patients and their families appeared to be multitask and covered the global wellness, both for patients and their families, and health care professionals, compliance to treatment prescriptions, psychological issues, and amelioration in quality of life (Banerjee et al. 2016; Vitale et al. 2021a, 2022). On the other hand, ineffective communication could ward off patients, recording higher levels in anxiety, depression, stress, job dissatisfaction, burnout (Donovan-Kicken and Caughlin 2011) uncertainty and dissatisfaction with care (Emold et al. 2011; Hagerty et al. 2005), increased lack of compliance with recommended treatment regimens, and elevated rates of depression and anxiety (Jin et al. 2008; Martin et al. 2005; Vitale et al. 2021a). Despite several advantages on effective communication among cancer patients and nurses and physicians, very few evidence are available reporting important obstacles and difficulties in communication benefits in their clinical settings. Recently, a clinical study has suggested two principal areas of communication like dealing with patients with bad news and their related emotional management (Pilsworth et al. 2014). Recent literature suggests new models to process questionnaires to highlight what participants say on their clinical practice (Nandwani and Verma 2021). Processing questionnaire items with predefined numeric answers or multiple-choice options is relatively simple, but extracting meaningful insights from open-ended responses remains a significant challenge due to the lack of standardized methods. While Natural Language Processing (NLP) techniques, such as sentiment and emotion analysis (Nandwani and Verma 2021), can offer some insights into the emotions of respondents, they often fail to capture the subtleties and nuances present in written language. Traditional NLP approaches typically identify basic sentiments like positive, negative, or neutral, and can detect emotions such as joy, anger, or sadness. However, these methods tend to oversimplify more intricate expressions of mood and intent, missing the richness of language found in open-ended responses.

The development of AI-driven Large Language Models (LLMs) (Wahlster 2023), such as OpenAI’s GPT and ChatGPT, has transformed the field of NLP, allowing for a deeper understanding of human expression. These models, trained on extensive datasets from a variety of sources, have the capability to grasp context, discern subtle shifts in tone, and produce coherent, contextually relevant outputs. LLMs offer significant advancements in sentiment analysis, enabling a more refined interpretation of written text. In addition to improving the quality of analysis, LLMs greatly enhance the efficiency of processing open-ended questionnaire data. By automating the extraction of insights, LLMs can handle large volumes of qualitative responses, reducing the need for labor-intensive manual analysis. This not only saves time and effort but also mitigates the risk of human bias and error, resulting in a more objective interpretation of the data.

In this study, we propose a hybrid approach that integrates traditional statistical analysis with the capabilities of LLMs to analyze and interpret structured and open-ended questionnaire responses, focusing on the communication of bad news in onco-hematology: health care professionals’ attitudes, communication methods, and perceived stress levels.

## Materials and methods

### Study design

An observational study was carried out from October 2023 to April 2024.

### Inclusion and exclusion criteria

All Italian physicians and nurses employed in an onco-hematology setting were considered as potential participants of our study. More specifically, physicians and nurses belonged to the “Italian Group for Bone Marrow Transplantation, Hemopoietic Stem Cells and Cell Therapy” (GITMO) and to “Noi delle Cure Palliative” social page were included, since the active link of the questionnaire was addressed.

### The questionnaire

The questionnaire was the same administered in our past research (Vitale et al. 2022). In this case, we administered the questionnaire only to physicians and nurses employed in onco-hematological settings, specifically to hematology and oncology both unit and day hospital settings, marrow transplant centers, pediatric and adult onco-hematology units, palliative care units and stem cell transplantation ones throughout the Italian territory.

The first part of the questionnaire collected sampling characteristics, such as sex, civil status, religious belief, work experience in oncology field, educational level, oncology setting, and job role.

The second part of the questionnaire contained items investigating self-perceptions on the interviewers’ attitudes on adoption of the SPIKE method in the bad news communication and related workplaces available to communicate bad news to cancer patients. Specifically, a total of 8 open-ended questions were proposed. Participants were invited to write brief and concise answers related to the following questions:

1. *What do you feel emotionally when communicating bad news?*
2. *How prepared do you feel when facing difficult communication situations?*
3. *How much do you think your empathy influences your ability to communicate bad news?*
4. *How much do you think your team’s support influences your emotional state during the communication of difficult news?*
5. *What aspects of the bad news communication process cause you the most stress?*
6. *What strategies do you use to relieve emotional stress after communicating bad news?*
7. *How do you evaluate your ability to manage your emotions during these conversations?*
8. *How do you perceive the effect of your communication on the emotional well-being of patients or families?*

Since this part of the questionnaire was created “*ad hoc*,” we firstly shared these items among Authors (O.P., E.V., R.L., L.C., and S.B.) to assess their comprehension thanks to the “Survey Instrument Validation Rating Scale,” which aimed to validate survey questionnaires [Oducado RM. Survey instrument validation rating scale, 2020. *Available at SSRN 3789575*]. A total of 13 items were proposed and each Author gave a preference associated to a Likert scale, as 1 for “Strongly Disagree,” 2 for “Disagree,” 3 for “Undecided,” 4 for “Agree,” and 5 for “Strongly Agree.” The items included in this validation survey were reported in Table 1.

**Table 1.** Validation rating scale by authors

Survey Instrument Validation Rating Scale	Author no. 1	Author no. 2	Author no. 3	Author no. 4	Author no. 5	Total score
The items in the instrument are relevant to answer the objectives of the study.	4	5	4	5	5	23
The items in the instrument can obtain depth to constructs being measured.	5	5	5	5	4	24
The instrument has an appropriate sample of items for the construct being measured.	4	5	5	5	5	24
The items and their alternatives are neither too narrow nor limited in its content.	5	4	4	5	4	22
The items in the instrument are stated clearly.	5	5	5	5	5	25
The items on the instrument can elicit responses which are stable, definite, consistent, and not conflicting.	4	5	4	5	4	22
The terms adapted in the scale in the scale are culturally appropriate.	5	5	5	5	5	25
The layout or format of the instrument is technically sound.	5	5	5	5	4	24
The responses on the scale show a reasonable range of variation.	5	5	4	5	5	24
The instrument is not too short or long enough that the participants will be able to answer it within a given time.	4	4	5	5	4	22
The instrument is interesting such that participants will be induced to respond to it and accomplish it fully.	5	5	4	5	4	23
The instrument as a whole could answer the basic purpose for which it is designed.	4	4	5	5	4	22
The instrument is culturally acceptable when administered in the local setting.	5	5	4	5	5	24

### Simple size

Considering Italian physicians, the National Federation of Boards of Surgeons and Dentists (into Italian: FNOMCeO) encountered 439,957 physicians (FNOMCEO. Osservatorio 2024). On the other hand, the National Federation of Associations of Nursing Professions (into Italian: FNOPI) in February 2024 encountered 279,837 nurses belonged to the Italian National Health Service, who were assigned in all medical wards (Ministero della Salute Direzione Generale della Digitalizzazione, del Sistema Informativo Sanitario e delle Statistica Ufficio di Statistica 2021). By Miller et al. (2003) formula, the representative sample encountered both 384 for physicians and nurses employed in all the medical specialties. The Italian Ministry of Health declared nearly 51 medical specializations (Direzione generale degli ordinamenti della formazione superiore e del diritto allo studio 2023). Thus, we aimed to reach nearly 50 nurses and 50 physicians employed in oncology and hematology facilities to reach a representative sample for our study.

### Data analysis

Data were collected in an Excel data sheet. Sampling characteristics were presented as frequencies and percentages. Considering open questions, the LLM was used.

### Open-ended questions analysis through Large Language Model

The qualitative data collected from the open-ended survey responses were processed and analyzed using LLMs to identify key themes and insights. This process followed the methodology outlined in a previous study (Lupo et al. 2024). Briefly, the analysis involved two main phases: first, vector embedding was applied to all textual responses, and then these vectors were clustered using the k-means algorithm, with the goal of detecting patterns within the data. In the second phase, the items in each cluster were summarized to provide a more detailed and synthesized overview of the emotions expressed.

We employed a freely available embedding model (Chia et al. 2023) to generate the embedding vectors, which were subsequently clustered into two groups, following an arbitrarily imposed partitioning scheme. The responses were categorized based on their thematic content. For example, answers to the question, *“What do you feel emotionally when communicating bad news?”* were grouped into two major categories: positive emotional responses and negative emotional responses. Responses that conveyed emotions such as empathy, calmness, and professional composure were classified as positive, while those expressing sadness, frustration, or powerlessness were assigned to the negative cluster. This approach was applied across all eight survey questions, allowing responses with similar emotional tones or themes to be grouped together.

After clustering, each group was analyzed to identify key emotions and reactions. To achieve this, important keywords representing central emotions or experiences were identified within each cluster. The frequency of these keywords was then calculated to determine the most common emotional reactions among the nurses. For instance, in the negative emotional responses to the first question, terms like “sad,” “powerless,” and “anxious” were frequently mentioned, whereas positive responses included words like “serene” and “empathetic.” This analysis helped us highlight the dominant emotional themes in each cluster.

Finally, each cluster was summarized to capture its main themes and insights. In the positive emotional response cluster, nurses typically expressed feelings of calmness, empathy, and professional awareness, suggesting a sense of control during difficult conversations. Conversely, the negative cluster revealed emotional strain, anxiety, and helplessness, indicating that delivering bad news can be a more emotionally taxing experience.

### Ethical considerations

The present study was approved by the GITMO trial office on January 15, 2024 that provided to disseminate the questionnaire through e-mails to all Italian nurses and physicians belonged to the GITMO organization. Additionally, we asked and then, obtained permission from “Noi delle Cure Palliative” social page who provided to spread the questionnaire throughout their subscribers.

The questionnaire respected both all the principles of the Declaration of Helsinki and the Italian data protection authority (DPA). It was emphasized that participation was voluntary, and that the participant could withdraw from the study at any time. Participant, who gave the informed consent, could complete the questionnaire. No data or alpha-numerical code were posted to guarantee the anonymity of the participant.

## Results

A total of 221 between Italian physicians and nurses employed in oncology and hematology settings were enrolled in the present study (Table 2).

**Table 2.** Participants’ characteristics (*n* = 221)

Participants’ characteristics	*n* (%)
**Sex**	
Female	118 (53.4)
Male	103 (46.6)
**Civil status**	
Unmarried	61 (27.6)
Married	112 (52.5)
Divorced/separated	38 (17.2)
Widower	6 (2.7)
**Religion**	
Christian	154 (69.7)
Atheist	37 (16.7)
Agnostic	27 (12.2)
Other	3 (1.4)
**Work experience in oncology settings**	
1−5 years	102 (46.2)
6−10 years	36 (16.3)
11−15 years	25 (11.4)
16−20 years	16 (7.3)
21−25 years	8 (3.7)
26−30 years	20 (9.3)
Over 31 years	14 (6.4)
**Educational level**	
Bachelor’s degree (3 years)	115 (52.1)
Master’s degree (3 + 2 years)	33 (15)
Degree in Medicine	7 (2.5)
PhD	61 (27.7)
Other	5 (2.4)
**Oncology setting**	
Hematology unit	42 (19.1)
Hematology day hospital	26 (11.8)
Oncology unit	33 (15)
Oncology day hospital	17 (7.8)
Marrow transplant center	25 (11.4)
Pediatric onco-hematology	33 (15)
Onco-hematology unit	17 (7.7)
Palliative care unit	28 (12.7)
**Job role**	
Physician	68 (30.8)
Nurse	153 (69.2)

Most of recruited participants were nurses (69.2%) and 30.8% were physicians. One hundred and eighteen participants were female and 103 males and most of participants (52.5%) were married; 69.7% of them declared to be Christian and the 46.2% of them worked in oncology settings less than 5 years.

The use of LLMs to analyze the open-ended responses provided valuable insights into the perspectives and emotional states of the respondents. We processed the responses to the eight open-ended questions from the questionnaire by applying vector embedding and clustering techniques. The results have been detailed in Table 3, presenting the keywords and their frequencies within the two identified clusters for each question, along with a concise summary of the main findings.

**Table 3.** Semi-automated analysis of responses to the eight open-ended questions. For each question, the table displays the keywords and their frequency in the two clusters, along with a summary of the corresponding results

Cluster	Keywords and frequency	Cluster summary
**Open-ended question 1:** What do you feel emotionally when communicating bad news?		
1	Serene (8), aware of my role (6), participative (5), empathetic (4), calm (3), professional (5).	This cluster represents positive emotions expressed by the nurses. The main emotions are empathy, serenity, professional awareness, and calmness. Health care professionals (HCPs) manage to maintain a serene or professional attitude while being aware of the role they play.
2	Sad (13), powerless (12), in difficulty (12), uncomfortable (8), demoralized (5), frustrated (4), devastated (4), anxious (3), sorry (3), sorry and distressed (1), worried and sad (6), anguished (1).	This cluster represents negative emotions. The main feelings include sadness, powerlessness, discomfort, and emotional difficulties. HCPs express anguish and stress in communicating bad news, reflecting a significant emotional impact.
**Open-ended question 2:** How prepared do you feel when facing difficult communication situations?		
1	Fairly prepared (21), prepared (3), ready (2).	This cluster represents feelings of preparedness. HCPs express confidence and readiness in facing difficult communication situations.
2	Unprepared (5), little prepared (10), not ready (3), difficult (2).	This cluster represents feelings of being unprepared. HCPs express insecurity, inadequacy, and lack of preparation in handling difficult communications.
**Open-ended question 3:** How much do you think your empathy influences your ability to communicate bad news?		
1	Very much/A lot (65).	This cluster represents feelings of high empathy. HCPs express that their empathy strongly influences their ability to communicate negative news.
2	Little (19), little to none (1), no influence (1).	This cluster represents feelings of low empathy. HCPs express that their empathy has little to no influence on their ability to communicate negative news.
**Open-ended question 4:** How much do you think your team’s support influences your emotional state during the communication of difficult news?		
1	Very much (82).	This cluster represents feelings of strong team support. HCPs express that team support significantly influences their emotional state during difficult communications.
2	No influence (8), little (13), not much (5).	This cluster represents feelings of low or no team support. HCPs express that team support has little to no influence on their emotional state during difficult communications.
**Open-ended question 5:** What aspects of the bad news communication process cause you the most stress?		
1	The difficulty of the patient and relatives in accepting the news (5), family members crying (3), not knowing the medical diagnosis and future developments (4), interlocutor’s response (2).	This cluster represents high-stress factors. HCPs report that the patient’s reaction, family members’ emotions, and the uncertainty of medical diagnosis contribute to higher stress during communication.
2	A little bit of everything (2), no particular source (4), collective stress (1).	This cluster represents low-stress factors. HCPs indicate that multiple aspects of the communication process cause stress, but without identifying a specific source of major stress.
**Open-ended question 6:** What strategies do you use to relieve emotional stress after communicating bad news?		
1	Live my life (1), hug (1), change the subject, distract on other topics (1), humor (1).	This cluster represents effective strategies. HCPs report that distraction, humor, and emotional support (such as hugs) are key strategies for alleviating emotional stress after delivering bad news.
2	Put oneself in the same situation (1).	This cluster represents less effective strategies. Some responses indicate minimal or ineffective coping mechanisms after delivering bad news.
**Open-ended question 7:** How do you evaluate your ability to manage your emotions during these conversations?		
1	Good (63), I can do better (1), good apparently in front of patients (1).	This cluster represents good emotional management. HCPs express confidence in their ability to manage their emotions, especially in front of patients.
2	I need to improve my human depth (8).	This cluster represents the need to improve emotional management. Some nurses acknowledge the need to enhance their emotional control during difficult conversations.
**Open-ended question 8:** How do you perceive the effect of your communication on the emotional well-being of patients or families?		
1	Very much (1), good (2), then patients show trust (1).	This cluster represents positive effects. HCPs feel their communication often fosters trust and is perceived positively by patients and their families.
2	It varies from patient to patient and from family to family (1), disorientation (1), medium-level (1).	This cluster represents negative or mixed effects. Some HCPs perceive that their communication can lead to disorientation or have a varied impact depending on the patient or family.

## Discussion

The application of LLMs to analyze open-ended responses provided valuable insights into the emotional experiences, preparedness, and coping strategies of nurses when communicating bad news. The results revealed a complex emotional landscape where positive and negative emotions coexist, with many HCPs expressing both confidence and distress depending on the context of the communication.

A significant number of respondents described feelings of empathy, serenity, and professional composure, which helped them maintain control during these difficult conversations. These professionals demonstrated resilience, often managing to balance their emotional involvement with the demands of their role. However, a substantial portion of HCPs reported experiencing emotional strain, with feelings such as sadness, powerlessness, and anxiety being prevalent. This emotional burden was frequently tied to the difficulty of patients and families in accepting bad news, as well as the uncertainty surrounding medical diagnoses. These findings are consistent with previous studies indicating that HCPs face emotional exhaustion and high levels of stress when tasked with delivering difficult news (Mitchell 2022; Moura et al. 2024).

In terms of preparedness, most nurses felt confident and well-equipped to handle difficult conversations. However, a notable group expressed feelings of inadequacy and a lack of readiness, pointing to a gap in training and emotional preparedness. This divide suggests the need for more structured support and education to help professionals develop the skills necessary to navigate these high-pressure scenarios, especially for those who feel unprepared or insecure. Previous research highlights similar gaps in training, particularly regarding how to manage the emotional and communicative challenges of breaking bad news (Nnate and Nashwan 2023).

One of the most critical factors influencing the emotional well-being of nurses during these interactions was the support they received from their team. HCPs who felt a strong sense of team support reported a more positive emotional state, which helped them manage the stress of delivering bad news. On the other hand, those who lacked such support often struggled with feelings of isolation and increased emotional strain. This underscores the importance of fostering a supportive work environment, where collaboration and emotional backing from colleagues can significantly mitigate the emotional toll of these difficult conversations (Biazar et al. 2022; Krieger et al. 2023).

Another key finding was the impact of empathy on communication. The majority of respondents indicated that their empathy greatly influenced how they communicated bad news, allowing them to connect more deeply with patients and families. Empathy was seen as a tool that enabled them to convey difficult information in a compassionate and sensitive manner. This supports the growing body of literature that emphasizes the importance of empathy in health care communication (Nnate and Nashwan 2023). However, some HCPs noted that empathy had little or no influence on their approach, suggesting that more procedural or task-focused methods were sometimes used instead. This diversity in communicative strategies points to the need for personalized training that respects individual styles while encouraging the integration of emotional intelligence into professional practice (Mitchell 2022).

When it came to coping with stress, HCPs employed various strategies to manage their emotional responses after delivering bad news. Effective techniques included distraction, humor, and emotional support from colleagues or loved ones, which were helpful in alleviating stress. However, some respondents indicated the use of less effective coping mechanisms, such as internalizing their stress by identifying too closely with the patient’s situation. These responses highlight the necessity of providing nurses with better tools and training in stress management to ensure they have healthy and effective ways to cope with the emotional demands of their role (Mitchell 2022; Moura et al. 2024).

Finally, HCPs’ perceptions of how their communication affected the emotional well-being of patients and families varied. While many believed their communication fostered trust and understanding, a few reported instances where patients or families experienced disorientation or reacted unpredictably. This variability suggests that while HCPs strive to provide compassionate and clear communication, the emotional impact of delivering bad news can differ widely based on individual circumstances, making it crucial to tailor communication strategies to each patient and family’s unique needs (Biazar et al. 2022; Krieger et al. 2023; Vitale et al. 2021b).

### Strengths and limitations

Surely, this study represented the first study investigating the bad news communication in oncology settings thanks the help of AI which allowed participants to introduce their thoughts and feelings without any close answer, but thanks to open answers they felt free to express their opinion.

However, the present study had several limitations. First of all, the questionnaire was administered into Italian. Answers and results were translated into English only to spread findings worldwide, not to validate the questionnaire. Then, the on-line nature of the questionnaire might limit the accessibility to participants to the questionnaire. Future studies will achieve to validate a tool in this field to better quantify strengths and limitations associated to the bad news communication.

## Conclusion

This study highlights the duality of emotions experienced by HCPs when delivering bad news – balancing professional composure with emotional distress. It underscores the critical role of empathy, team support, and adequate preparation in helping nurses navigate these challenging conversations. However, the findings also reveal gaps in training and support systems, pointing to the need for more robust interventions to help nurses develop both the emotional and communicative skills required to manage these high-pressure situations effectively, as also reporting in previous studies (Katz 2019; Wittenberg-Lyles et al. 2013).

## Data Availability

Data sharing is not applicable to our article.
