# Communicating bad news in oncology and hematology settings: A statistic and Large Language Model for interpreting nurses’ difficulties and emotions – CORRIGENDUM

**DOI:** 10.1017/S1478951526101886

**Published:** 2026-03-17

**Authors:** Elsa Vitale, Luana Conte, Marco Cioce, Patrizia Cornacchione, Angela Capuano, Raffaella Massafra, Ludovica Panzanaro, Giorgio De Nunzio, Matteo Steduto, Chiara Visintini, Sara Errichiello, Alfonso Parisi, Gabriele Sperti, Olga Pomes, Roberto Lupo, Stefano Botti

**Affiliations:** 1 Scientific Directorate, IRCCS Istituto Tumori Bari Giovanni Paolo II, Bari, Italy; 2 Department of Physics and Chemistry, University of Palermo, Palermo, Italy; 3 Advanced Data Analysis in Medicine (ADAM), Laboratory of Interdisciplinary Research Applied to Medicine (DReAM), University of Salento and ASL (Local Health Authority) Lecce, Lecce, Italy; 4 Department UOC SITRA, Fondazione Policlinico Universitario Agostino Gemelli IRCCS, Rome, Italy; 5 UOC Fisica per le Scienze della Vita, Dipartimento di Diagnostica per Immagini e Radioterapia Oncologica, Fondazione Policlinico Universitario Agostino Gemelli IRCCS, Rome, Italy; 6 Department of Emergency, AORN Santobono-Pausilipon, Naples, Italy; 7 C.R.A.P. Carrubo, Sol Levante srl Avetrana, Taranto, Italy; 8 Laboratory of Biomedical Physics and Environment, Department of Mathematics and Physics “E. De Giorgi”, University of Salento, Lecce, Italy; 9 Haematology Intensive Care Unit, Fondazione IRCCS Casa Sollievo della Sofferenza, San Giovanni Rotondo, Italy; 10 Haematology and Stem Cell Transplantation Unit, Udine University Hospital, Azienda Sanitaria Universitaria Friuli Centrale, Udine, Italy; 11 Division of Hematology and Bone Marrow Transplant, Fondazione Istituto di Ricovero e Cura a Carattere Scientifico, Istituto Naziona, Milan, Italy; 12 Department of Hematology and Bone Marrow Transplant, Hospital Card. G. Panico, Tricase, Lecce, Italy; 13 University Hospital Consortium of Bari, Bari, Italy; 14 San Giuseppe da Copertino Hospital, ASL (Local Health Authority) Lecce, Lecce, Italy; 15 Hematology Unit, Azienda USL-IRCCS of Reggio Emilia, Reggio Emilia, Italy

**Keywords:** Bad news, communication, generative artificial intelligence (GAI), oncology, hematology, setting, corrigendum

The Author apologises for incorrect author affiliation information which was published in the article for these three authors.

Patrizia Cornacchione – changed to affiliation 5

Angela Capuano – changed to affiliation 6

Ludovica Panzanaro – changed to affiliation 7 only.

These are now correct in the published article.
